# Institutional Notes

**Published:** 1902-02-01

**Authors:** 


### INSTITUTIONAL NOTES.

AT Guy's Hospital there were accommodated last year 7,811 in-patients. In the casualty and out-patients' departments 120,000 were treated, and care was given to 4,000 maternity cases.

IN the circular placed in the church pews of Aberdeen on Sunday, January 5th, 1902, the following particulars of work done by the Aberdeen Royal Infirmary during the past year were given, viz., Number of in-patients, 2,714; out-patients, 4,842; casualty cases, 4,633.

IN the fourth report of the committee of the Victorian Convalescent Home for Surrey Women at Bognor, it is stated that the amount invested for the endowment fund now reaches at cost, £11,086 2s. 11d., and produces an annual interest of approximately £350.

---

At the annual meeting in connection with the Hospital Sunday and Saturday movement, Wigan, Mr. Davies moved that the amounts of £386 16s. 2d. and £51 2s. 10d. be transferred to the credit of the maintenance account of the Royal Albert Edward Infirmary and Dispensary. This was agreed to.

---

An entertainment in aid of Charing Cross Hospital new nursing home will be held in the Ball Room of the Savoy Hotel in the afternoon and evening of February 10th and 11th. The leading attractions will be tableaux specially designed and arranged by Messrs. George Frampton, A.R.A., J. J. Shannon, A.R.A., and J. M. Swan, A.R.A.

---

At a meeting of the committee of the Aberdeen University Extension Scheme held on January 3rd, it was announced that they had raised the sum of £29,942, and that in fulfilment of the promise made a year ago Lord Strathcona had sent his cheque for £25,000 to make up the amount required for the completion of the buildings.

---

A new home for the accommodation of nurses in connection with the Manchester and Salford Sick Poor and Private Nursing Institution has been opened at Harpurhey. There is accommodation for twelve nurses and the working staff, and the total cost has been £5,000, of which £1,600 has yet to be obtained. The land and £500 were the gift of Mr. T. Berry.

---

In a circular issued from the Northern Counties Hospital for Consumption and Diseases of the Chest, Newcastle, the committee state that an offer has been made to them by a well-known citizen to give a donation of £100 and an annual subscription of £20 to £25 (provided £900 more in donations can be raised), in order to start a sanatorium for the open-air treatment of consumption. The committee therefore trust that a ready response will be made to this appeal.

---

A joint isolation hospital for Chelmsford and the surrounding rural district will shortly be an established fact. The Mayor of Sheffield, in proposing that the scheme be approved, said that it was very inadvisable that there should be two hospitals under two separate managements, as there had been up to now, and that it was for the welfare of both parties that there should be only one hospital. Alderman Taylor seconded the resolution, which was carried unanimously.

---

The Duchess of Buccleuch and Queensberry, who is president of the Scottish branch of the movement for the "Further Endowment of Queen Victoria's Jubilee Institute for Nurses," is appealing to her countrymen and women to assist in raising the sum of £30,000 to permanently wipe out the annual deficit of £600 which has been accumulating for some years. The honorary secretary of the movement is Miss Guthrie Wright, 29 Castle Terrace, Edinburgh, who will be pleased to furnish any further particulars that may be desired.

